# Clinical Predictive Modeling of Heart Failure: Domain Description, Models’ Characteristics and Literature Review

**DOI:** 10.3390/diagnostics14040443

**Published:** 2024-02-17

**Authors:** Igor Odrobina

**Affiliations:** Mathematical Institute, Slovak Academy of Science, Štefánikova 49, SK-841 73 Bratislava, Slovakia; igor.odrobina@gmail.com

**Keywords:** prediction, model, heart failure, telemedicine, prognosis, diagnosis, detection, monitoring, characteristic

## Abstract

This study attempts to identify and briefly describe the current directions in applied and theoretical clinical prediction research. Context-rich chronic heart failure syndrome (CHFS) telemedicine provides the medical foundation for this effort. In the chronic stage of heart failure, there are sudden exacerbations of syndromes with subsequent hospitalizations, which are called acute decompensation of heart failure (ADHF). These decompensations are the subject of diagnostic and prognostic predictions. The primary purpose of ADHF predictions is to clarify the current and future health status of patients and subsequently optimize therapeutic responses. We proposed a simplified discrete-state disease model as an attempt at a typical summarization of a medical subject before starting predictive modeling. The study tries also to structure the essential common characteristics of quantitative models in order to understand the issue in an application context. The last part provides an overview of prediction works in the field of CHFS. These three parts provide the reader with a comprehensive view of quantitative clinical predictive modeling in heart failure telemedicine with an emphasis on several key general aspects. The target community is medical researchers seeking to align their clinical studies with prognostic or diagnostic predictive modeling, as well as other predictive researchers. The study was written by a non-medical expert.

## 1. Introduction

Digital data are currently available in abundance in all healthcare facilities. Once automated analysis and prediction systems are built, researchers could realize a complete real-time analytical and decision support system. The widespread presence of electronic health records (EHRs) is also changing clinical prediction and analytical research. The new possibilities of data-driven research are pointed out in [1].

Modern applied and theoretical clinical prediction research bridges medicine, statistics, machine learning (ML) and engineering. All these areas have their own methods and terminology. A researcher trying to understand this field must become familiar with the minimum basics in these fields.

In order to gain a representative sample of already applied predictive models, we focused on the well-studied topic of telemedicine care for patients with chronic heart failure syndrome. We were driven by expectations that this approach, with some modifications, would form the framework for identifying cutting-edge topics and procedures in clinical predictive modeling. We found that a wide variety of statistical and machine learning models have been used in this area. In order to grasp the topic in its entirety, we have divided the study in accordance with the title into three parts.

In the first part, basic information about chronic heart failure syndrome and its telemedicine was compiled primarily from medical journals. In addition to this, we aimed for a systematized description of CHFS and other diseases allowing the deployment of quantitative models. The answer we propose takes the form of disease-specific UML-style modeling consisting of discrete disease states with descriptions of the transitions between them. This type of modeling appears to be a needed abstraction layer between accepted quantitative models and the body of medical knowledge. In literature, analogous modeling is considered the basis for primarily prognostic predictions. However, when we investigated the time-unfolded version of this model, we found that it closely corresponds to the known schematic of CHFS disease progression. We consider this schematic as the continuous counterpart of the time-unfolded discrete-state disease model. Its continuous nature provides the foundation for early diagnosis of ADHF decompensation. We consider observed correspondence to be an indication of the viability of the model development approach.

Quantitative models are part of an abstraction layer that is more distant from the body of medical knowledge than the disease-specific models mentioned above. Understanding their characteristics allows the researcher to mutually compare the predictive properties of the available models, such as those reviewed in the third part of the study, and consider their new clinical applications. In the second part of the study, we try to encompass these quantitative models into a structure consisting of a single mathematical or algorithmic core “surrounded” by its external determinants, speaking in figurative manner. These determinant elements are prediction inputs, prediction outputs, ability of prognostic or diagnostic predictions and some other properties of prediction models. The traits of the determinants are relatively independent of the inner core of the quantitative prediction model, so we will call them here *common characteristics* of quantitative models. These common characteristics are of primary concern to clinical trial designers, and they strongly determine the overall utility and benefit of the prediction.

Special attention is given to the diagnostic process in telemedical settings. Patient monitoring includes repetitive and frequent partial diagnostics of the early stages of ADHF decompensation and we propose to name this type of diagnostic process *recurrent diagnostics.* The process can be used for a timely and optimal therapeutic response. We seek to develop a determinant-based model classification framework to aid current prediction efforts to optimize predictive classifiers for higher performance.

The classification of prediction models, but in a different way, was made in [2,3] and general modeling recommendations were formulated in [4,5]. In this study, we do not deal with the methodology of model construction and deployment. The details of the calculation procedure and machine learning algorithms are also perceived to be of secondary importance here.

The primary purpose of the third part is to provide an overview of statistical and machine learning models with special emphasis on heart failure syndrome and the specifics of diagnostics in telemedicine settings. The review can provide a starting point for predictive research, modification of existing research, and complete model redesign based on predictive approaches used in other areas of medicine. After completing the review of publications on the prediction of CHFS, we expanded the literature search to include additional directions. First, it was the direction of the *early warning systems* used in emergency rooms (ERs) and intensive care units (ICUs) that were selected for time-repeated diagnostics analogous to that in CHFS telemedicine. Another direction concerned the subsection dedicated to advanced statistical models used mainly in the prediction of other types of diseases. The last direction was related to the subsection describing additional modeling approaches. This was conducted in an attempt to enable an understanding of the theoretical limits of the predictive ability of the models reviewed before.

### 1.1. Content and Structure of the Study

The study was organized as follows. In the Section 2 we present an introduction to the clinical basis of chronic heart failure syndrome and the telemedicine of the syndrome. We describe the syndrome and its decompensation denoted as ADHF from a medical and modeling point of view. These subsections are followed by a description of telemedical patient remote monitoring. Section 3 provides the reader with a general description and guidelines for medical prediction research. Section 4 presents a classification of prediction models according to their outer characteristics. In it, we elaborate on topics such as the object of prediction, the form and timeline of predictive information, timelines of target and predictor data, and groups and types of prediction data. We discuss prognostic and diagnostic alternatives to model focus. At the end of the section, we discuss the machine learning approach in relation to traditional statistical modeling. Section 5 first provides an overview of remote monitoring with attempts to perform diagnostic or detection prediction of decompensations. Next, it provides a reference to several review studies that prognostically predict patient decompensations. This is followed by a subsection describing advanced statistical models and other statistical models. At the end of the section, we summarize prognostic and diagnostic predictions carried out by machine learning researchers. Section 6 and Section 7 provide the closure of this study.

### 1.2. Literature Search Method

The publication database MEDLINE represents an overwhelming variety of medical topics and directions. In our opinion, no single review study can fully satisfy a reader interested in a particular field.

In order to meet the objectives formulated in the abstract, we tried to conduct our searches as cross-sectional as possible. Our literature review selects only a few publications from a particular research direction that we hope are the most recent or the most comprehensive. We are not trying to conduct a complete review of any specific direction. The most numerous articles on predictive modeling are those in which the modeling is based on data present in the EHR. They usually predict ADHF decompensation and hospital readmission as a prognosis for CHFS. These prognostic predictions are mainly based on biomarkers, one-time medical examinations and demographic data.

Not many reviews have been published on the topic of detection or predictive diagnosis of ADHF in telemedical settings. This prediction is based on data that are collected with a relatively high repetitive rate and the data constitutes a special kind of EHRs.

In the field of advanced statistical models, we found only a few articles dealing with the prognosis of CHFS yet, but we present these models for a more complete review. We included two publications dealing with the prognostic prediction of CHFS incidence or *incident heart failure*.

Our review is based on searches performed primarily in the MEDLINE database via the PubMed search engine. The results were confronted with the findings in Google Scholar. We consider the scope of the topic under investigation to be so diverse that we have not attempted to cover our findings within some unifying search query and search scheme. Naturally, in the early stages of the search, we created a complex search query that included words such as forecast, model, heart failure, telemedicine, or remote monitoring, but later we focused on other search methods, such as snowballing through article links.

## 2. Medical Domain Description

The content of this section was compiled by a non-medical expert and is intended for modeling purposes.

### 2.1. Chronic Heart Failure Syndrome and Its Decompensation

Chronic heart failure syndrome is a frequent and long-term disease that burdens the patient’s life and represents a burden on the medical care system. The syndrome arises as a result of various worsened underlying cardiovascular health conditions. In general, after the appearance of typical signs and symptoms, comprehensive examinations of CHFS are performed before the diagnosis is finally confirmed [6,7] (in our age of powerful computerized translators also [8] might be considered). CHFS is often grouped into two categories according to the status of left ventricular ejection fraction (LVEF). Heart failure in patients with reduced LVEF is referred to as HFrEF, and in patients with preserved LVEF as HFpEF. Patients are further classified according to the NYHA scale and the score of the KCCQ-12 questionnaire. Many patients progress to a stage called *advanced heart failure* characterized by persistent symptoms [7].

In addition to the slow continuous deterioration of the quality of life, the patient’s life is disrupted by a sudden worsening of symptoms, which is called *acute decompensation of heart failure* or ADHF. For simplicity, in this work, we will adopt the terminology used in [6]. In the European guidelines [7] there is a term *acute heart failure* or AHF and acute decompensation is referred to as only one of four different types of acute presentations. In addition to acute decompensation, the other three presentations are acute pulmonary edema, isolated right ventricular failure, and cardiogenic shock.

We could now say that our broader definition of ADHF now includes four distinct presentations [9] with different temporal characteristics of progression. We will discuss the implications of these issues in the modeler’s subsection. The differences in terminology mentioned above were addressed in [10].

At the end of this section, we could mention that the worsening of symptoms requiring hospitalization as the beginning of CHFS is called *de-novo acute heart failure* [9,11]. It is a separate topic and we do not deal with it here. If one wants to understand the extent of the medical and biochemical models of CHFS, one should look at the works of D. L. Mann et al. [12].

### 2.2. Disease-Specific Prognostic and Diagnostic Models

In addition to biochemical modeling, two additional modeling processes exist related to the CHFS prediction task. The first additional modeling process tries to grasp the anatomy of the disease and the diagnostic-prognostic issues. The person performing this activity should be called a medicine *domain expert* and should describe the investigated problem in some form of modeling language such as UML. The second modeling process refers to quantitative predictive modeling, where a mutual combination of predictors and a mathematical formula provides the value of the predicted parameter. The person performing this modeling process is a statistical or machine learning expert. In the study, we do not always strictly distinguish between these two versions of modeling.

The modelers do not necessarily need to know every detail of the biochemistry of the investigated health condition, but they need to know the basics about timelines, predictors, manifestations and all possible outcomes of the disease. They should also be aware of the fact that ADHF decompensation is a relatively autonomous biomedical pathological sub-process of CHFS with a more or less well-defined onset and end. It would also be useful to know whether ADHF is triggered by some random cause (external or internal), or whether decompensation occurs as a natural internal progression of CHFS. A list of probable random causes triggering ADHF decompensation and their statistics are given in [13] (we should note that we have not found much independent support for these observations in literature yet).

It seems reasonable to concentrate compiled medical knowledge about a disease on some disease-specific diagrams or models. As we will see later, all the main points for this are already present in the literature. First, we present a model for heart failure syndrome, which is the primary prognostic one. Next, we present a schematic of CHFS from an experienced research team that we propose to represent a disease-specific model for diagnostic purposes in telemedical and telemetric settings. The diagnostic process under these settings we call *recurrent diagnostics*. This model pair stands between medical knowledge and the purely quantitative, disease-independent models described in Section 4.

**Figure 1:**
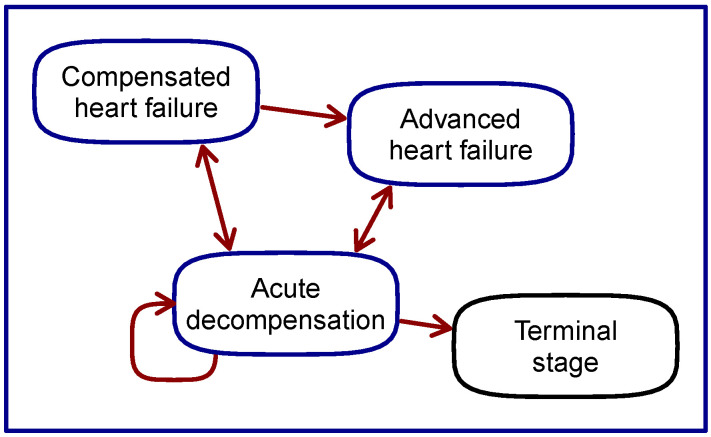
Diagram of states and transitions of chronic heart failure syndrome. Red straight arrow indicates state transition. Curved arrow indicates a state recurrence. The terminal stage refers to the patient’s irreversible progression to death. We are aware of the possibility that the transition between the state of compensated HF and the advanced state of HF is well defined only through the state of acute decompensation. The proposed model of HF can be subjected to further modifications.

The health information about CHFS can be summarized in the form of a four-state diagram in Figure 1. To the basic three states (compensated state, acute decompensation state and terminal stage) we added a fourth state, the advanced heart failure state in accordance with [7]. We did so because this state differs significantly in a diagnostic, and probably also prognostic, sense from the compensated HF state. For example, according to ([7], Chapter 4), the weight of the patient in the compensated and advanced state develops in the opposite direction, which may indicate a significantly different disease state not only from a diagnostic, but also from a prognostic point of view. A change in the patient’s body weight is a key sign of heart failure syndrome. Ignoring the existence of advanced heart failure state in modeling might be responsible for lower prognostic predictivity of hospitalization, as mentioned in ([6], Chapter 4).

This is the disease modeling approach advocated by Houwelingen [14] and others. In their model formulation, transitions are associated with probabilities or rates. These models are primarily suitable for prognostic quantitative predictions. It should be noted that the acute decompensation state does not have the so-called *memoryless* property (the concepts of state memorylessness and probability-characterized state transitions are present in Markov chain (MC) modeling). This is related to the fact that the ADHF state is preferentially restored to the state from which it originated. ADHF can also recur, and then the patient is re-hospitalized within a month or two of discharge.

**Figure 2:**
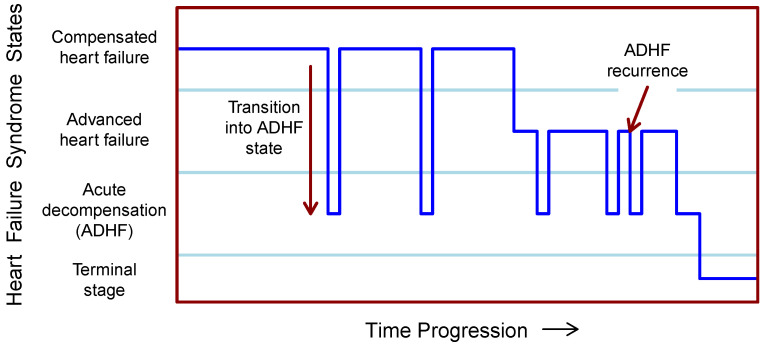
Illustration of the progression of chronic heart failure syndrome as a sequence of time-varying states. The light blue horizontal lines mark the boundaries of the four syndrome states. Dark blue line indicates patient’s state. Transitions between states are depicted as instantaneous.

The state diagram in Figure 1 can be unfolded into a temporal progression of CHFS states, as shown in Figure 2. The first transition from the compensated state to the decompensated ADHF state is explicitly marked. Towards the end of the diagram, ADHF recurrence is illustrated.

The Figure 1 and Figure 2 together can represent a model of the disease states and events in a patient with chronic heart failure syndrome. All prognostic studies reviewed in Section 5 are based on simplifications of this model into two-state forms. Compensated and advanced heart failure states are merged together and published studies focus separately on transitions to acute decompensation and on transitions to the terminal stage. In the latter case, the ADHF state is supposedly a subpart of the terminal stage.

**Figure 3:**
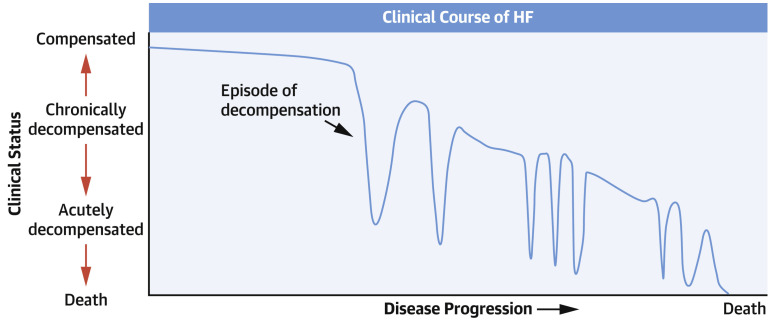
Depiction of the heart failure syndrome course as a development of clinical status. Source: Januzzi and Butler [15]. Reproduced with permission from Elsevier.

In Figure 2 we see that when the states of the syndrome are arranged by disease severity on the vertical axis, a striking correspondence with Figure 3 reprinted from [15] appears. Although the dark blue line in Figure 2 represents the patient’s time-varying states, the diagram in Figure 2 resembles some form of discretization of the patient’s clinical status curve in Figure 3. The similarity is even stronger when we take into account that the disease state *Advanced heart failure* corresponds in a clinical sense to the label *Chronically decompensated* on the left axis. This correspondence is particularly remarkable when we consider that Figure 2 was produced in accordance with EU guidelines and Figure 3 was very likely produced in accordance with US guidelines.

We might perceive the depiction of the disease in Figure 3 by a clinical status curve also as basis for a disease model but of a different type. The clinical status curve in Figure 3 could probably be developed so that the curve represents a disease indicator, such as that in [16]. The indicator could be carefully engineered as a combination of the patient’s diagnostic parameters or in other ways, and this could be diagnostically highly predictive. As we will show later, such a model may be capable of diagnostic prediction modeling and diagnostic prediction of ADHF under telemedical or telemetric settings. We can thus come to the conclusion that Figure 3 can be considered as a graphic representation of a self-contained disease model of substantial diagnostic relevance. We should note that the authors of [15] do not make any particular claims about their diagram, and the text of their article interprets the Figure 3 more or less as a kind of convenient depiction of the CHFS disease development.

To summarize Figure 1, Figure 2 and Figure 3, we can say that Figure 1 represents a discrete-state disease model for prognostic prediction, Figure 3 with the hypothesized explanation above, represents an important type of disease model for diagnostic prediction under telemedical or telemetric settings, and the unfolded time progression of disease states in Figure 2 shows a background agreement between these two types of models.

In prognostic and diagnostic prediction, it is important to have clear specifications of the outcome events, or manifestations of transitions to target clinical states. The basic ones can be an irregular visit to the ambulance, hospitalization, or death. Each type of event can have its own optimized set of predictor variables. As previously mentioned, a patient admitted to the hospital with ADHF may have four quite distinct clinical presentations [7]. Neglecting this variability in modeling can negatively impact model performance. As an example, authors of the work [17] show that in the prognostic prediction of new-onset heart failure syndrome, the exact outcome specification has a significant impact on the selection of optimized predictors of the final model.

The modelers, especially in diagnostic prediction, should understand the underlying temporal characteristics of the predicted acute process. There may be several subtypes of outcome events, and the onset of the event can be gradual (days) or rapid (hours), or indeterminate ([7], Chapter 11). It might be beneficial for them to have an idea of the nature of the symptoms and the main clinical manifestations. In the case of a controlled trial, this may affect the selection of the optimal set of measured medical parameters. In the case of a retrospective study, clinical knowledge is also important at least to eliminate the presence of outliers and systemic outliers.

At this point, we should address the existence of insufficiently clear boundaries between key medical concepts. In this work, we will simply assume that there is no transition period between the compensated stage of CHFS and the acute decompensated stage (ADHF). This assumption is in apparent contrast to the designation expressed in the title of the important and cited publication [18]. We associate the transition period mentioned in the title with some early stages of the acute decompensation process. These early stages are manifested, for example, by changes in the patient’s pulmonary arterial pressure.

At the end of the subsection, we mention that the understanding of the field of CHFS is hindered by the fact that the global medical community follows two rather different systems of reasoning, characterized by two separate guidelines [6,7]. The modelers should also be aware that the syndrome is characterized by non-specific symptoms and signs [19], that there is no single test to establish the diagnosis of CHFS [20] and that 14–29% of cases are misclassified even after examination in the emergency room [21].

### 2.3. Telemedical Remote Monitoring of Patients with Heart Failure

Modern telecommunication technologies have also penetrated the field of health care for patients with chronic heart failure syndrome. These technologies make it possible to use a hitherto unused set of data describing the patient’s signs and symptoms, which are collected during the ordinary life of the patient on a daily basis or even more often. These data have remained unused until now despite its importance [22]. The importance of collecting this type of data in the home environment is also documented by the CHFS guidelines [7], which says, for example, that if the patient’s weight increases above a certain level over a certain period, the therapist or the patient himself should administer an increased dose of diuretics.

Medical staff in telemedicine trials now have unrestricted access to this daily data in parallel with the patient’s biomarkers and medical examinations obtained during initial or regular visits. The therapist now has the opportunity to use them to adjust their actions in order to ensure the best long-term prognosis for the patient. It should be noted that the primary role of remote patient monitoring in CHFS telemedicine is to improve patient medical management; decompensation prediction is only a subset of this primary assignment. The authors [23] hypothesize that *“it seems plausible that the most potential treatment effect of telemedicine (TM) comes from a more optimal use of diuretics and the up-titration of heart failure (HF) medication”*. The optimization of medication doses based on CHFS telemedicine data was investigated in [24].

As can be seen in the review by [25], before 2002, telemedicine data were collected in a non-invasive way, i.e., without any wearable devices and implants. Currently, the number of projects using invasive methods of remote monitoring of patients with CHFS is growing. Recently, review articles [26,27,28] attempted to evaluate the overall impact of implant-based telemonitoring on the management of patients with CHFS. It should be noted that distrustful views have also been expressed about this technology [29]. Wearable devices in this context have been investigated in [30].

On the other hand, in addition to invasive and device-assisted methods, there are still many new non-invasive telemonitoring studies in chronic heart failure medicine. A survey [31] found that CHFS telemonitoring was associated with a 20% reduction in all-cause mortality and a 37% reduction in CHFS hospitalization. Other CHFS telemedicine trials were reviewed in [32,33,34,35]. We could consider the work of [36] as the most promising study of non-invasive telemonitoring, which shows the positive benefits of telemedicine care above a statistically significant level. It is not self-evident that this outcome can be achieved, and many other telemedicine studies [37,38,39,40] show that CHFS telemedicine improves patient outcomes, but not as much as required by the 5% level of statistical significance.

The common characteristic of telemetry data is the relative simplicity of their monitoring, their collection is often conducted by the patient himself. The term *vital signs* usually refers to heart rate, blood pressure, respiratory rate, and body temperature, but we prefer to use the term more loosely as a category of data collected at a high repetition rate that also includes weight change, and oxygen saturation level. We will return to this issue later in the discussion of grouping data types.

## 3. Clinical Prediction Models in General

Clinical prediction and clinical prediction tools are an integral part of modern medicine. A large number of prediction models are published every year. The basics of predictive modeling in medicine are summarized in [5]. Chapters aimed at a medical audience have been included, such as “Predictive Modeling Studies”, “Predictive Model Applications”, and more. Systematic evaluation of the clinical utility of predictive modeling is a complex task and requires a decision and analytical framework [41]. Another team of authors evaluated the impact of prediction models in [42]. More work of this kind is needed to clarify the medical foundations of prediction research and to overcome the doubts that have been directed at it like in [43].

Given the diversity and complexity of the prediction research community and prediction research itself, there are also efforts to guide the research and reporting process by specifying a fixed set of rules. Intuitive and disorganized reporting of developed models can very easily devalue the primary achievements and messages of the authors. Therefore, a joint effort to structure and regulate the issue of model reporting appeared. An initiative called “Transparent reporting of a multivariable prediction model for individual prognosis or diagnosis” or the TRIPOD [44,45] came into existence, in which the basic principles are explicitly formulated. Methodological guidance for models’ updating can be found in [41].

It is well known that models are often subject to bias. Another initiative emerged and developed the “Prediction model Risk Of Bias Assessment Tool” or PROBAST tool [46,47]. The tool consists of four fields: participants, predictors, outcome and analysis. These domains contain a total of twenty signaling questions to assess the risk of bias. The level of risk of bias generally depends on the study design, conduct and analysis. A high risk of bias indicates a significantly distorted performance of the model’s predictive output.

Very valuable information about predictive modeling and the properties of statistical models can also be obtained through area-specific guidelines [48] and systematic reviews [49]. A practical guide to clinical prediction modeling can be found in [2].

## 4. Common Characteristics of Quantitative Prediction Models

The objective of this part is to provide a structured, unified view of quantitative predictive models in statistics, engineering, and machine learning. These fields attempt to solve the prediction task defined in the medical domain of chronic heart failure syndrome, where the situation is captured by the disease model condensed in Figure 1 and Figure 2. We consider the introduction of this view as an analogy to the introduction of an additional type of diagram when describing a domain problem in UML language.

**Figure 4:**
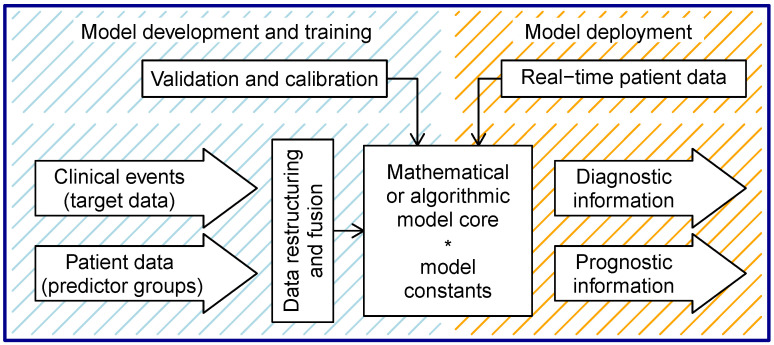
Schematic representation of the clinical quantitative modeling and model deployment.

As we have already mentioned, prediction models can be assessed according to their external elements or characteristics. External model elements or characteristics can be introduced as features of the model that do not belong to the internal statistical or algorithmic core. They represent a kind of surroundings of the model core interior. They are shown in the lower part of the Figure 4. Of all the characteristics present in the model, in this part, we focus on the object of prediction, the time characteristic of diagnostic and prognostic information, target and predictor data, and types and groups of prediction data. The set of model characteristics also includes information on whether the model deals with prognostics or diagnostics and whether a statistical core or a machine learning core was used. At the end of this section, we present two simple examples of mathematical model cores.

In order to explain the relationship of this section to Section 5, we must state that the elements or characteristics listed here are also intended to provide an underlying quantitative modeling framework for the prediction models reviewed later in the aforementioned section.

Before continuing, we would like to remind non-mathematicians that the concept of probability or risk of developing a disease can be imagined as the proportion of materialized positive cases within a relevant cohort of patients in a time interval. The next discussed *hazard rate* can then be understood as this probability divided by the mentioned time interval.

### 4.1. Characteristic #1—Object of Prediction

A prediction is a statement about a clinically relevant issue that is in a state of uncertainty at the moment of prediction. The concept of mathematical probability is used to quantitatively express prediction. In clinical practice, there is uncertainty about the presence of the disease or its stage at the moment of prediction. A prediction can also be a quantitative probability statement about the occurrence of a disease or its stage in the future.

In the context of already diagnosed CHFS, the focus of prognostic and diagnostic predictions shifts to the occurrence of worsening symptoms, the appearance of a stage of decompensation with admission to the hospital, or the occurrence of death. These are all visible manifestations of a sudden change in the compensated CHFS state. The primary aim of this study is to investigate the prognostic and diagnostic prediction possibilities of ADHF in patients with an already established diagnosis of CHFS who are under telemedicine monitoring. The model review part also includes works with prognostic predictions of deaths.

### 4.2. Characteristic #2—Prediction Information Timelines

#### 4.2.1. Diagnostic Information Timelines

Prediction can be aimed at predicting the presence of a disease or its stage in a patient at the current moment. This is a diagnostic prediction. The meaning of the word prediction seems to be related primarily to the uncertain nature of the prediction statement. Diagnostic uncertainty fades over time in two ways. The first is related to the timeline of disease progression when the disease manifests itself with more intense and visible symptoms. The second is connected to the timeline of the sequence of diagnostic steps when more accurate and unambiguous tests are applied later in the sequence.

In the first case, the signs and symptoms of the disease or its new stage are detected and the diagnosis is predicted. The validity of the prediction is confirmed by the explicit manifestation of the disease only with a certain time delay, which is clearly shorter than the duration of the entire pathological process, which in our case is ADHF. Diagnosis or detection of ADHF by measurement of pulmonary arterial pressure may precede hospital admission by approximately twenty days [18]. The certainty of diagnostic prediction is quantitatively expressed by the values of sensitivity and specificity (quantitative definitions of sensitivity and specificity are given in Table A2) when larger values mean greater certainty. The quality of the entire diagnostic method is assessed by the Area Under the Receiver Operating Characteristic (AUROC) curve.

In the field of CHFS telemedicine, the diagnostic procedure is carried out remotely regularly with a high frequency of repetition. The moment of diagnosis moves forward, and as hospitalization approaches, the certainty of diagnosis should change towards higher values.

In the second case, in the case of a sequence of diagnostic tests, the prediction is refined by applying more accurate additional tests. The therapist makes a decision about the disease not only on the basis of a more accurate test but also considers the results of the previous ones. The issue of combining information from several diagnostic tests or symptoms is of fundamental importance, and its mathematical description is discussed in Section 4.7.

#### 4.2.2. Prognostic Information Timelines

The presence of ADHF is manifested by the event of the patient’s admission to the hospital. The decompensations are said to occur randomly, so they are manifestations.

There are two distinct types of prognostic prediction of decompensation in the literature. The first type of prognostic prediction is the prediction of the occurrence of decompensation in the near and distant future. The second type concerns only the near future, which means that the time interval for the rate or probability calculation starts from the moment of prognostic prediction.

During the modeling process, the prognostic period corresponds to the entire period of the follow-up study. The prognostic period should be much longer than the typical duration of decompensation.

For the first type of prognostic prediction, the powerful concept of *hazard rate function* [50,51,52] is widely used. The hazard rate or frequency of decompensations in a patient cohort may change over a relatively long prognostic period. This is why the time-dependent function is used to capture the prognostic information as a whole.

A precise definition of the hazard rate function can be made through its relation to the probability of an event or probability of change in the disease state denoted *P*. Mathematically, it can be expressed as follows. First, the randomness of a disease event is described by a random variable *T* which represents the time of occurrence of the event. The hazard rate function h(t) is then defined as the rate of occurrence of events at time *t*. Time *t* is positive and less than or equal to the prognostic period. Using the formalism of probability equations, this can be expressed as [50,51,52]:

(1) $$h\left(t\right)≃\frac{P(t\leq T<T+\Delta t|T\geq t)}{\Delta t}\;,$$

where Δt is the interval for counting events to obtain an observational estimate of the probability *P* and should be long enough to eliminate statistical noise. The Δt is not directly related to the duration of the decompensation process (ADHF) but must be reasonably longer than the duration of its manifestation (e.g., the duration of the hospital admission acceptance process). It is usually much shorter than the prognostic period.

In the context of CHFS prognosis, the expression (1) reads that the hazard rate h(t) is the rate at which patients in the cohort experience the occurrence of decompensation. The condition T≥t in the conditional probability says that the calculation of the proportion takes into account only those patients who did not experience the event and were not censored until time *t*.

The hazard rate function can be constant, increasing, U-shaped, or shaped in some other way, as shown for example in [53]. To obtain a sense of the possible statistical noise distortion of the observed hazard rate functions, one should look at the examples in ([50], Chapter 2). The Kaplan–Meyer, Nelson–Aalen, and Cox models with their variants are used to calculate the hazard rate function. The hazard rate value obtained for the whole cohort can be individualized according to individual patient characteristics, as discussed in Section 4.7. The quality of the prognosis can be evaluated using time-dependent receiver operating characteristic (ROC) curves [54,55].

If the patient undergoes repeated outpatient or inpatient examinations during the illness, additional data sets with additional prognostic capabilities are created. Prognosis can be repeatedly reassessed and the result is a multiple set of hazard functions. This prognostic situation is systematically addressed by the methods in Section 5.3. As a prediction result, a fragmented hazard function or otherwise compounded hazard function is obtained.

The second, simplistic type of prognostic prediction is a prediction for the near or impending future. The prognostic period corresponds to the counting interval Δt and a constant value of the hazard rate is assumed. The interval can be as long as a day, a week, a month, a year, or even as long as the patient’s remaining life. The outcome events are counted together during the entire follow-up period. In the context of CHFS, the number of outcome events represents the *cumulative incidence* of decompensations. During this period, the group of patients is partially reduced, but the period can be chosen short enough not to significantly affect the modeler’s quantitative predictions. The cumulative probability of events *p* is calculated in the interval Δt and has the form of a simple equation:

p(Δt)=P(0≤T<Δt|T≥0),

where *T* is again a random variable assigned to the time of the event. When the time interval Δt is reasonably short, the relationship between the cumulative probability *p* and the hazard rate function h(t) can be expressed using approximate equality:

$${p\left(\Delta t\right)≃h\left(t\right)|}_{t=0}\;\Delta t\;.$$

The approximate equality can provide a quick estimate of the hazard rate when the proportion of the cumulative incidence of events is less than 10–20% of the total number of patients. The well-established logistic regression is widely used in this type of prognostic prediction.

### 4.3. Characteristic #3—Temporal Properties of Target and Predictor Data

#### 4.3.1. Temporal Properties of Target Data

Target data represent basic information about recorded clinical events. In a simple diagnostic prediction model, the data need not have explicit temporal characteristics. If a continual diagnosis of the monitored patient is performed, the target data can be bounded by a sliding time window that moves with the moment of diagnosis.

In case we are building a model specifying the prognostic hazard rate function, we need to have event time data in the data set. They are present there in the form, e.g., that a patient event record contains the patient ID, event time, and event type coded into a categorical variable. The role of this target *time-to-event* data in models is significantly different from the role of time data specifying the time of the predictor value. A simpler, previously defined second type of prognostic prediction model does not require the precise specification of the time of the event. The length of the follow-up period, which is equal to the length of the counting interval Δt, is sufficient.

#### 4.3.2. Temporal Properties of Predictor Data

Incorporating time dependence into predictor variables seems to be one of the primary challenges of prediction models in contemporary prediction research. We call statistical models that directly include the time dependence of predictors advanced models due to a significant increase in their complexity.

First, in the simplest case, the predictor variables have no significant time dependence at all. Predictor data are collected over a time period of negligible length. In the context of CHFS research, this is the case of a patient’s entry into a clinical trial or case of a hospital entry examination to confirm ADHF diagnosis. Over the course of the clinical trial, data are not updated, and information about the patient’s ever-changing vital signs and symptoms are ignored or not collected. This time-free data are termed as *cross-sectional* data.

The second case occurs when a patient visits a therapist during a clinical trial and their biomarker and other data are updated on a quarterly or monthly basis. These data usually contain a time dependence, but the data update frequency is relatively low. These episodic or regular visit data enter the prediction models in a significantly different way than the target time-to-event data. These data are called *longitudinal data*. Models using this type of predictor data are summarized in Section 5.3 and Section 5.4.

The third case occurs when vital signs and disease symptoms are recorded and actively incorporated into the modeling in a telemedicine clinical trial. These types of data are collected at a significantly higher repetition rate compared to the previous case. In telemedicine or home patient care settings, these data are collected daily or almost daily. For vital ICU signs, the collection rate can be hours or even minutes. The term longitudinal data is rarely used in the literature for these data, and the term *time series data* seems to be preferred. In telemedicine, these data are used in predictive models to diagnose the coming acute decompensation, or in other words to detect the early stages of ADHF. In the intensive care unit, these data are used to probabilistically determine, e.g., the 24-h risk of adverse events such as cardiac arrest.

#### 4.3.3. Temporal Properties of Recurrent Diagnostics

In telemedicine and ICU settings, the diagnostic or detection processes of incoming decompensation or other adverse events take place repeatedly, and the diagnostic process differs from others. We proposed the use of the term recurrent diagnostics. In this prediction setting, the timeline enters the prediction model in three different ways. The first way is the time dependence of prediction inputs, the second way can be time as a parameter of prediction result and the last way is that time enters the model as a repeatedly shifting moment of the act of diagnostic prediction.

During the determination of the sensitivity and specificity of the diagnostic method, the target input data represent the delayed explicit manifestations of the disease. The determination requires a certain time interval to compile target data to confirm or disprove the validity of the disease prediction. We can call the chosen time interval *forward target window*, (the descriptiveness of the names assigned to temporal windows depends on the reference context. As an example, in a similar situation the authors in [56] use a different notation) and it should be large enough to cover the mentioned manifestation delays. In the field of CHFS telemedicine, the essence of patient monitoring is a process of recurrent diagnostics. The moment of diagnosis is constantly shifting in time, as is the beginning of the forward target window.

Another relevant time interval appears in the predictor data. This interval contains temporal changes and temporal patterns important for predicting an impending event. When monitoring a patient recurrently, it is natural to include multiple records from the recent past. They could also serve to eliminate random noise from recordings. These previous data are again part of the diagnostic prediction process and can be considered as part of another time window, which we could call the *retrospective predictor window*. This window also shifts with the progress of the diagnostic moment. The two prediction windows mentioned above could together be termed as *sliding time windows* [57]. The introduction of similar windows is also present in other works and in the field of recurrent diagnostics, it represents an additional form of input data structuring.

**Figure 5:**
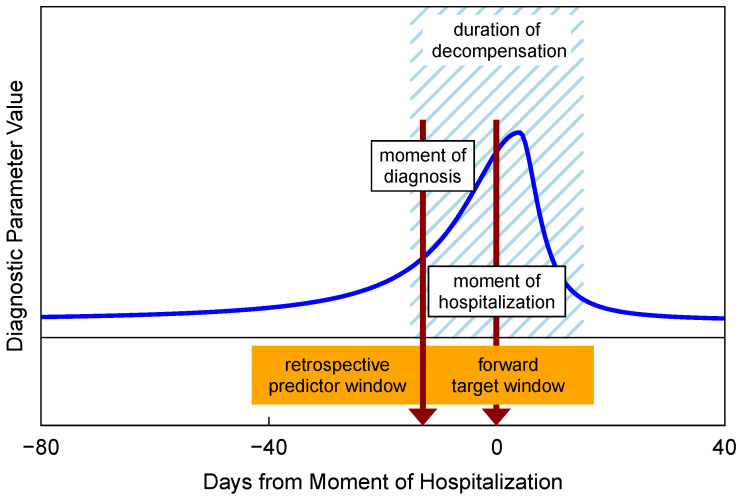
Schematic of recurrent telemedical diagnostics against the background of patient decompensation (hatched area). The illustration of the diagnostic parameter development (blue line) is made in accordance with [16,59].

The relation of both windows to the development of diagnostic parameters is shown in Figure 5. The schematic describes a retrospective modeling situation, so we know with certainty that in this case the hospitalization event definitely occurred. We can rescale the time axis so that the moment of hospitalization corresponds to time zero. The retrospective predictor window specifies the range for the predictor data, which are plotted in the figure by the blue line. The forward target window determines the range of the target data. The only target value in the scheme is represented by the act of hospitalization at time zero. The moment of diagnosis is marked with a dark red arrow. Figure 5 represents a more general view of recurrent detection-diagnostic prediction process investigated, e.g., in [58].

Provided that the clinical status parameter in the suggested heart failure model in Figure 3 is linked with the patient’s diagnostic parameters, their model has the ability to clarify the diagnostic processes of acute decompensation (ADHF) during telemedicine monitoring. The overall picture of the telemedicine recurrent diagnostics of heart failure patients can be obtained by gradually superimposing the inverted diagnostic parameter hump in Figure 5 (blue line) over the pronounced depressions on the patient’s clinical status curve in Figure 3.

### 4.4. Characteristic #4—Processing of Different Types and Groups of Predictor Data

The timelines for a typical telemedicine-controlled trial are as follows. The telemedicine study begins with an entrance examination of both the control and intervention groups. The study continues with telemedicine monitoring of the intervention group, which may last half a year or longer. Telemedicine data of the intervention group are collected in the home environment daily or almost daily. Data on regular and episodic visits to the ambulance are also stored. At the end of the clinical trial, both groups will undergo a final exit examination and the results will be used for comparison. According to the temporal characteristics, the data can be classified into several groups and two subsections, as shown in the Table 1.

A fundamental aspect of these data groups is their heterogeneity in relation to time. In the upper part of the Table 1 we can see that some groups of data are collected only once or twice, another group of data is collected episodically or with a low frequency (monthly, quarterly), and some data are collected with a high repetition frequency (daily). While entry examinations, demographics, and comorbidity data serve primarily for prognostic purposes of ADHF and death, high-frequency signs and symptoms are critical for diagnosing ADHF. Low-frequency data are primarily used for prognostic purposes and are used mainly by methods presented in Section 5.3

In addition to the data groups listed in the upper part of the Table 1, there are two other important groups of clinical factors that influence the therapist’s prediction and decision-making and are also significant in quantitative prediction. The first type is the possible presence of *“risk-changing clinical milestones”* [60]. For example, the dates and number of hospitalizations significantly affect prognostic and, indirectly, diagnostic predictions. The second important group of predictors is the types of drugs used and their dosage. In the case of CHFS, certain types of drugs strongly influence prognostic prediction and have also been selected by medical experts as part of prognostic scores such as SHFM and MAGGIC [61,62]. Diuretic dosing affects changes in patient body weight, and therefore, can strongly influence the diagnostic prediction of ADHF. In addition to drug dosing, there may be other influential therapeutic interventions that also need to be considered. Neglecting these additional factors can lead to poor performance of all types of prediction methods.

#### 4.4.1. Increasing Predictive Power by Combining Heterogeneous Groups

To make a suggestion on how to deal with the various groups of data in Table 1 we take a look into the area of the well-established early warning systems used in ERs and ICUs. There are obvious similarities between these systems and our ADHF telemedicine diagnostic system and the heterogeneous data types are present in these systems too. The combination of several different groups of predictors with different temporal characteristics into one prediction process has been labeled as a data fusion ([1], Chapter 22). The authors build on predictive modeling works [63,64] and order their data into groups in the manner shown in the Table 2. The data fusion method can also be considered as a technique of combining primarily prognostic data types with diagnostic ones. By developing the presentation of Table 2, we kept the data structure similar to the original one, but the medical parameters were changed to correspond to the CHFS area. The presented sets of CHFS trial data types are for modeling purposes, they are compiled from literature and project proposals by non-medical experts.

Comparing Table 2 with the upper part of Table 1, we see that the final examination data group and the low repetition frequency data group are missing. Nevertheless, we believe that the ideas of the data fusion technique are also applicable to telemedicine trials.

In [65], a Bayesian Belief Network (BBN) methodology is used to combine different datasets to increase the discriminative power of predictors in telemetry settings. Baseline data were treated as prior probabilities, and BBN probability tables were used to calculate the posterior probability of HF decompensation. The authors also propose a BBN-based discriminator capable of providing a recommendation for different therapies. The proposal seems to be mainly about electronic stimulation therapy of the implant device, but the idea may apply more generally. The baseline group and the signs-and-symptoms group are also combined in [66] to diagnose ADHF in a telemedicine environment.

#### 4.4.2. Other Methods of Increasing Prediction Ability

For a better prediction success rate, we could compose new predictor variables that could contain information about the time derivative or time integral characteristics of the originally observed predictors [67]. Basic statistics literature ([50], Chapter 8) recommends creating these new variables as well. As an example, the relative time derivative of the observed variable is created. This new variable served to capture time dependence in prediction variables in a standard Cox proportional hazards model.

The term *feature engineering* is used in both the engineering and machine learning communities for the process of creating new, directly unobserved variables. Publications [68,69] present a list of engineered variables from telemedically monitored daily data of patients with CHFS in order to diagnose ADHF.

Monitored telemedicine prediction data can be processed to create a predictive alert signal. In [58,70], an extended moving average method called MACD is used to generate a warning signal from a single monitored variable such as body weight. The pattern similarity principle is used to generate an alert signal from monitored patient vital signs in [57]. The predictive ability of individual signals can be strengthened by combining them with each other using the naive Bayesian assumption [68,69,71,72].

### 4.5. Characteristic #5—Distinction between Prognosis and Diagnosis

A natural start to understanding the distinction between the terms of prognosis and diagnosis is to follow the timelines of these predictions. Prognosis deals with the situation where the pathological process of the disease is predicted to occur sometime in the future. Diagnostics deals with the current situation and assesses whether the disease process has started or not. We could repeat the statement in [41] that *“clinical prediction models are tools that predict health outcomes either at present (diagnostic) or in the future (prognostic)”*. The difference between these two prediction categories is also described in [44,73].

In more complex situations such as continuous patient monitoring of ADHF, the above distinction is inconvenient to clarify the situation. We prefer to use differences in clinical parameters. In the classification schematic [73], the authors underscored the observation of the presence of disease signs and symptoms as the predominant difference between diagnosis and prognosis. In the case of diagnosis, we can rely on the presence of signs and symptoms of the disease, while in the case of prognostic prediction we cannot do this because the patient does not yet have the predicted state of health. For prognostic prediction, we should rely only on other patient clinical parameters, such as biomarker values, clinical examination results, etc.

One should not be confused by applying schematics in [73] for prediction classification in our CHFS field. In the schematics, the authors use the term cross-sectional to describe the process of diagnosis. However, the authors use the term to describe the simultaneity between the moment of the latest prediction data and the moment of the predicted state of health. This may cause some confusion because the term “cross-sectional” is often associated with predictor variables, and this type of variable is regularly used in prognosis.

**Figure 6:**
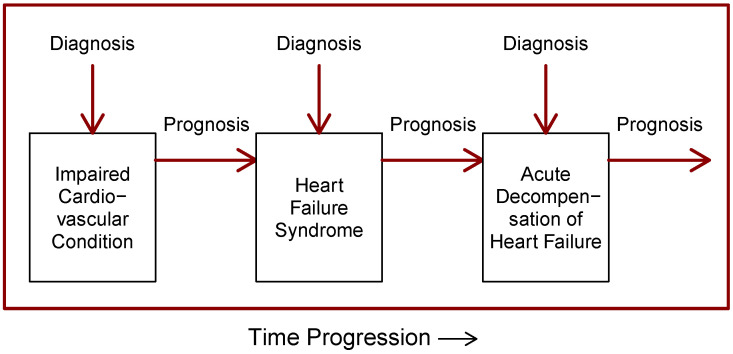
History of diagnoses and prognoses in a patient with ADHF.

It should be noted that in situations when it comes to a disease with a long and complex medical history, we are dealing with a relatively long sequence of prognoses and diagnoses. A diagram of the diagnostic-prognostic sequence undergone by a patient in the acute decompensated stage is shown in Figure 6. We see that at the beginning, an impaired cardiovascular condition occurred and was diagnosed. Within the prognosis of impaired cardiovascular condition, there is a possibility that chronic heart failure syndrome may occur. Once the CHFS occurs and is diagnosed, the prognosis of the syndrome is that a decompensated state of ADHF may follow. In telemedicine monitoring, ADHF is pre-diagnosed (or detected) in an outpatient setting, followed by a confirmatory diagnosis of ADHF in a medical facility. Again, the ADHF state has its prognoses, such as recovery to a compensated state, recovery to a chronically decompensated state, readmission relapse, and unfortunately, death.

Another term, the detection, is associated in the literature with the act of predicting a medical condition. It may come from the authors’ engineering background as a convenient substitute for the term diagnosis [57,69,71]. However, the term detection also seems to be used in situations where the use of the term diagnosis is not easily applicable. This seems to be the case with early warning systems [74,75].

In this study, when predicting impending acute decompensation, we prefer the term ADHF diagnosis and follow the use of the term, e.g., in [76]. We prefer to comply with the recommendations formulated in TRIPOD [44] and in [73].

### 4.6. Characteristic #6—Statistical Approach versus Machine Learning

The discussion on the relationship between the statistical approach and the machine learning was started by L. Breiman’s article with valuable comments that express the position of several recognized statisticians [77]. Statistical approaches are based on a solid theoretical data model and the idea of likelihood in the background. Statistics also have imperfect models called *working models*. Mathematics is also part of machine learning. One must admit that there is a certain similarity between the search for maximum likelihood in statistics and the minimization of the error function in the field of neural networks. However, machine learning seems to be trying to build a perfect algorithm that provides perfect responses in response to input data, rather than building a perfect data model in the background.

In the area of prognostic survival modeling, these two approaches have been summarized [3] in understandable model hierarchies. Recently, machine learning has attracted critical attention from researchers with a medical background. In the area of ADHF prediction, a critical appraisal of ML studies was presented in [78]. Studies [79,80] address the issue of performance and reliability of machine learning models within a broader clinical perspective.

Despite the criticism, it should be emphasized that machine learning modeling represents a fundamentally different approach by researchers from different backgrounds. In theory, their challenges could prevent clinical prediction research from stiffness in methodology and concepts. On the other hand, it seems that machine learning researchers do not pay due attention to the fundamentals of clinical prediction formulated in the TRIPOD statement and PROBAST tool requirements.

Machine learning researchers have brought new concepts to the field of clinical prediction research that are not well established in statistics. Some of it comes from their engineering and especially their software engineering background. The term *domain expertise* draws attention to the fact that there is a relatively large area between modeling and medicine that does not belong to either modelers or medical professionals. Another term *conceptual embedding* describes the process of mapping clinical terminology to universal modeling concepts. In our opinion, this assignment deserves a clear name. Concepts used by healthcare professionals are formed by clinical practice and require additional specifications before being used in quantitative modeling. Probably the most intriguing is the introduction of the concept of *feature engineering*, which expresses the fact that modelers are not limited by the form of observed data and are encouraged to use their modifications as predictive variables. Machine learning experts introduced these concepts, probably because the application domain in their field often changes and this requires persistent flexibility.

### 4.7. Mathematics of Quantitative Models: Basic Prognostic and Diagnostic Tasks

Mathematics is present in all prediction models and plays a key role in model formulation and application. In the following text, we present a mathematical approach to two main problems in clinical prediction research.

#### 4.7.1. Basic Prognostic Model

As an example of mathematical prognostic prediction, we present the Cox proportional hazards model. The Cox model is widespread; it has become a sub-model in prognostic joint models [81] and a second stage in so-called two-stage models such as landmarking [14]. It has many variants and extensions [51]. It is challenged only by a model called discrete time logistic regression [74] developed in [53]. The original logistic regression compares the Cox model only when the cohort decline is not significant and the hazard rate function can be approximated by a single value. It should be noted that if cohort attrition is the only concern, a stepwise Cox method can be used, such as in [61].

The following is not intended to compete with the explanations of the Cox model available in the current literature [50,51,52], but merely to provide a tangible example of a hazard rate function for the interested reader. The Cox proportional hazards model expresses individualized hazard rate functions from the statistics of the entire clinical trial cohort. The basic model input is that there are *n* patients indexed i=1,2,…n and each patient has *p* clinical parameters. The parameters of the *i*th patient can then be denoted as $x_{ji},j=1,2,\ldots p$. These are the values that are recorded when a patient enters a clinical trial. The expression for the individualized hazard rate function $h_{i}\left(t\right)$ for the *i*th patient has the form [50]:

(2) $$h_{i}\left(t\right)=h_{0}\left(t\right)\exp(\beta_{1}x_{1i}+\beta_{2}x_{2i}+\ldots +\beta_{p}x_{pi})\;,$$

where $\beta_{1},\beta_{2},\ldots \beta_{p}$ are constants determined by the modeling process. Patient characteristics $x_{ji}$ may be the result of their clinical tests or may represent their demographic data. The function $h_{0}\left(t\right)$ is the *basal hazard rate function*. The adjective basal means that it provides reference values for all individualized hazard rate functions. In the Cox proportional hazards model, the ratio between the values of the patient’s hazard rate function and the basal hazard rate function does not change over time. The ratio is fully determined by the model constants β and values of patient characteristics $x_{ji}$. The sum of the values of the predictor $x_{ji}$ multiplied by the coefficients $\beta_{j}$ seen in Equation (2) is called the *linear term*. Determining the values of the β coefficients is a key modeling issue and the topic is discussed in Appendix A.

Patients’ overall risk is often expressed as a patient’s *risk score*. There are different ways of expressing its value, the SHFM risk score given in [61] seems most appropriate for the subsection describing the Cox model. The patient’s risk score is expressed there in a very convenient way; the risk score called SHFM is simply the linear part of the Equation (2). Other ways of defining risk scores can be found in the literature.

#### 4.7.2. Basic Diagnostic Model

The widespread availability of electronic health records makes it easy to conduct quantitative research on diagnostic procedures. A review of published sensitivities and specificities of symptoms for the diagnosis of ADHF was conducted in [21]. As we mentioned earlier, ADHF is characterized by symptoms of low specificity, and therefore, the issue of combining diagnostic information from more than one symptom or sign is important.

From a mathematically exact point of view, the therapist performs a set of diagnostic steps where the initial posterior probability of the presence of the disease is constantly replaced by new improved posterior probabilities under new evidence. The mathematical explanation and formulation of the problem is as follows. The probability $P_{1}\left(D|E_{1}\right)$ of the presence of a disease state *D* at the result of the first diagnostic step $E_{1}$ can be expressed by Bayes’ theorem in perhaps the most transparent form as:

(3) $$P_{1}\left(D|E_{1}\right)=\frac{P(E_{1}|D)}{P\left(E_{1}|D\right)P_{0}\left(D\right)+P\left(E_{1}|\neg D\right)P_{0}\left(\neg D\right)}\;P_{0}\left(D\right)\;,$$

where $P_{1}\left(D|E_{1}\right)$ represents the posterior probability of the disease, the expression $P_{0}\left(D\right)$ is the probability of the disease state *D* in the population. In case *D* indicates the presence of the disease, the expression above represents the prevalence of the disease (for further explanation see Table A2). The term $P(E_{1}|D)$ is the probability of the test result $E_{1}$ on the disease state *D*, $P(E_{1}|\neg D)$ is the probability of the test result $E_{1}$ on the inverted disease state.

We consider the form of the Equation (3) to be transparent because in this form we can pair it with its clinical interpretations [82,83]. A detailed medical interpretation of the equation can be found in Appendix B. When the second diagnostic step $E_{2}$ is performed, the probability of the presence of the disease in the patient changes to:

(4) $$P_{2}\left(D|E_{2},E_{1}\right)=\frac{P\left(E_{2},E_{1}|D\right)P_{0}\left(D\right)}{P\left(E_{2},E_{1}|D\right)P_{0}\left(D\right)+P\left(E_{2},E_{1}|\neg D\right)P_{0}\left(\neg D\right)}\;,$$

where the pair $\left(E_{2},E_{1}\right)$ represents the state of the *combined test*. The term $P_{2}\left(E_{2},E_{1}|D\right)$ is the probability of the result of the combined test $\left(E_{2},E_{1}\right)$ conditional on whether the disease *D* is present or not. $P_{2}\left(E_{2},E_{1}|\neg D\right)$ is an analogous probability, but under the condition that the inverted disease state is taken into account. Aspects of combining two diagnostic tests are described in detail, e.g., in [84].

A tempting approach is to simplify the Equation (4) by assuming that the combined test $\left(E_{2},E_{1}\right)$ is the set of two independent tests $E_{2}$ and $E_{1}$. The assumption of independence often referred to as *naive* would transform Equation (4) into the form:

$$P_{2}\left(D|E_{2},E_{1}\right)=\frac{P\left(E_{2}|D\right)P\left(E_{1}|D\right)P_{0}\left(D\right)}{P\left(E_{2}|D\right)P\left(E_{1}|D\right)P_{0}\left(D\right)+P\left(E_{2}|\neg D\right)P\left(E_{1}|\neg D\right)P_{0}\left(\neg D\right)}\;,$$

where $P(E_{2}|D)$ and $P(E_{2}|\neg D)$ are the probabilities of the second test result $E_{2}$ depending on the disease state *D* and ¬D, respectively. If test independence is assumed, then it is possible to formally calculate the positive predictive value of these combined two tests with knowledge of the individual sensitivities and specificities of the tests and the prevalence of the disease. Unfortunately, this simplifying assumption, if not well substantiated, will lead to misleading results in a large number of cases, and the results obtained should not be considered valid.

## 5. Overview of Heart Failure Prediction Models

The assumption of all the following prediction models is that the diagnosis of CHFS has already been made in the patient. We would like to repeat that all the publications reviewed here do not deal with de-novo acute heart failure [9,11,85]. All reviewed models assume already diagnosed chronic heart failure syndrome (CHFS) and predict acute decompensation (ADHF) or death. The prediction of new-onset heart failure or incident heart failure is also a separate topic and these models are reviewed in [86].

### 5.1. Telemedical and Telemetric Diagnostic Prediction of Acute Heart Failure Decompensation

We consider that the primary purpose of these works is early detection or diagnosis of acute decompensation (ADHF). Prediction models can be distinguished according to the criterion of whether an invasive or non-invasive method was used in relation to the patient. The prediction object is the early stage of ADHF. The earliest stage of detection for diagnosis is achieved by measuring the increase in pulmonary arterial pressure [18]. We consider, as already mentioned, that the pathological process of decompensation can occur several weeks before it is clearly manifested by the admission of the patient to the hospital.

#### 5.1.1. Published Samples of Telemedical and Telemetric Data

Weight change due to fluid retention is considered the most important predictor variable in the CHFS telemedicine monitoring system. Considerable work has been devoted to this matter of fact. To obtain a sense of the weight change of a patient before and after hospitalization with ADHF, one should review the real data or their averaged profiles, which can be found in [58,69,87,88]. Daily intrathoracic bioimpedance data in the post-discharge period are presented in [89]. The daily dependence of intrathoracic impedance before and during hospitalization is presented in [90]. Implanted devices have also been used to collect daily data from CHFS and other types of cardiac patients. Their characteristics and averages are listed in [16,59].

#### 5.1.2. Non-Invasive Prediction Methods

Among the first attempts to create a predictive model of the clinical deterioration of a CHFS patient is work [91]. Data were collected using a patient weight record book. Zhang et al. [58] used a classification method originating from the financial industry called MACD. In relation to the input data structuring introduced in Section 4.3.3, we should say that their forward target window size was chosen to be 14 days; the optimized retrospective window size for the predictors was found to be 80 days. The method used, at least in our opinion, is capable of good prediction of the upcoming stage of CHFS deterioration, despite the authors’ skepticism about the method. Their work influenced later works.

There is a brief review of non-invasive ADHF detection models included in [71] and will not be repeated here. To enlarge their list of models, we present three more. The first one is AHDF prediction using wavelet transform [57]. Their predictive recurrent detection method is based on a sliding window approach and pattern identification. The development of four different predictor variables was used—and four different pairs of sensitivities and specificities were obtained. These four parameters were collected daily, body weight, blood pressure, heart rate and respiration rate. The second work we would like to add to the list is [69], where a number of proposed features based on a single time-varying variable were tested as a basis for physiological signal detection and ADHF diagnosis. To achieve better alert signaling performance these signals were merged using a naive Bayesian method and this was used in their ADHF prediction system [72]. The last non-invasive ADHF prediction method we will mention is the work of [56]. The performance of Bayesian online change point detection (BOCPD) and retrospective change point detection (RCPD) methods was evaluated. The former was better for events with a rapid onset, the latter for events that have slower gradual changes. In the Discussion section, the authors present a brief overview of approaches in their own and related fields.

#### 5.1.3. Prediction Models Using Implants

The companies Medtronix and Optivol are known for integrating patient monitoring devices into implantable cardioverter-defibrillators and similar devices. The patient’s clinical parameters were monitored by various sensors. Initially, the threshold zone method [92] was used and later the Bayesian belief network [65,93] was used to combine the sensor signals to obtain a decompensation prediction. Their method was named TriageHF™ risk score [94]. A concurrent effort in predicting ADHF decompensations was made by Boston Scientific [95]. Patients had to have an implanted defibrillator for cardiac resynchronization therapy. Their HeartLogic™ multisensor index and alert algorithm provides a sensitive and timely predictor of impending ADHF decompensation. Details of their signal evaluation can be found in [96,97]. Both implant-based prediction technologies have been comprehensively analyzed and evaluated in [76].

#### 5.1.4. Confirmatory Diagnostics of ADHF

The above methods provide us with only a preliminary diagnostic indication of decompensation. This prediction is followed by a detailed examination in a medical facility. Even then, the diagnosis of ADHF is not completely certain. The issue is addressed by a systematic review of sensitivities and specificities of various diagnostic parameters in [21].

### 5.2. Prognostic Prediction with Cross-Sectional Predictors

Prognostic tools of this type in the treatment of CHFS are recommended in guidelines for the management of heart failure ([6], Chapter 4). They should be used both for the prognosis of death and for the prognosis of hospitalization, but the effectiveness differs. A brief overview of the tools is also provided in there.

A survey of statistical models was carried out in 2008 [98]. During the study period (1988–2007), multivariate logistic regression and Cox proportional hazards regression were mainly used. Less than 15% of the publications use the $\chi^{2}$ test only. An analogous survey was repeated in 2022 [78]. Similarly, Cox regression, logistic regression, and score methods were considered typical statistical models. In addition to statistical models, the authors also investigated machine learning models and we will mention them later. The review [99] also includes statistical models and machine learning models, the review conducted in [100] can also be noted.

Prognostic information about the risk of decompensation or death is often encapsulated in a simple scoring system. The developed scores vary in performance and have been compared in many publications. A comparison of the popular SHFM risk score and the MAGGIC score can be found in [101]. The SHFM score [61] is the linear part of a stepwise Cox proportional hazards model, where the model has been adjusted to use a constant hazard rate function. The MAGGIC [62] score is a converted Poisson regression model predictor. For further risk score performance comparisons see, e.g., [102,103].

### 5.3. Advanced Statistical Modeling with Time-Dependent Predictors

The modeling situation becomes unexpectedly complicated when the time parameter appears not only as an event parameter but also as a part of the predictor variable. In that case, two different timelines appear. The first comes from the time of events and the associated data are called *time-to-event data*. The second timeline is from when the predictor characteristics (e.g., biomarkers) were collected and the associated data are usually called *longitudinal data*. There are three basic approaches to dealing with this situation from a statistical modeling point of view [104,105]:

(i) a naive approach—simply use the obtained longitudinal data as predictors in models such as the Cox proportional hazards model,
(ii) two-stage modeling approach where longitudinal predictors are addressed first and time-to-event data are incorporated later. The most used model of this class seems to be the *landmarking model* [14], a generalized landmark model was recently introduced in [60],
(iii) true *joint model* approach, which consists of two models coupled by sharing random effects [81,104,106].

There is literature that has compared the advantages of the landmarking approach and joint modeling [105]. Comparison by simulation is conducted in [107,108]. These models are used for prognostic predictions, but so far we found only a few articles dealing with chronic heart failure syndrome [109,110].

### 5.4. Other Advanced Statistical Models

The following are a group of prediction models for one to be aware of, but which do not belong in any of the previous subsections. The first type of model includes the phenomenon of long-term changes in the entire population and in the health care system. This phenomenon results in a temporal and spatial shift of the model constants. Models need to be recalibrated and the effect of the change is called *calibration drift*. An overview of these models is given in [111]. Ref. [112] approaches the topic of model calibration in general.

Incorporating time-varying coefficients into the Cox model is considered an extension of it in [51,113,114]. An alternative to obtaining the hazard function by a model like the Cox model was demonstrated in [53]. The model is presented in the review of early warning systems [74] and is labeled there as *discrete time logistic regression*.

### 5.5. Machine Learning Approaches

Machine learning techniques (often referred to as artificial intelligence) also enter the field of clinical prediction modeling of CHFS. The application of machine learning methods to CHFS syndrome is freshly reviewed in [115,116,117]. It has become an excellent practice to compare the efficiency of machine learning classifiers with the efficiency of established and well-researched logistic regression. We should note that recently the performance advantages of machine learning methods over traditional methods have been reviewed [79]. In the following, we will divide our brief overview of machine learning in the CHFS area into two parts.

#### 5.5.1. Machine Learning for ADHF Detection and Diagnosis

Short-term prediction of hospitalization using a similarity-based machine learning (SBM) method was performed in [30]. Patients used a single wearable device during the clinical trial. The used positive window was 10 days long and corresponds to our forward target window. As a side note, the authors claim in the abstract that they have developed a prognostic algorithm to detect CHFS exacerbation. In this study, we would consider their prediction as part of the diagnostic process of decompensation, and we would prefer to call the algorithm a diagnostic detection algorithm.

The performance of seven machine learning methods was compared with the performance of logistic regression in [118]. The retrospective predictor window was assumed to be seven days, the forward target window was also chosen to be seven days. The Boruta method was used to eliminate insignificant predictors. The authors concluded that, among other methods, the extreme gradient boost (XGBoost) method performs in the best way.

The performance of the long short-term memory network (LSTM) was compared with logistic regression and the multi-layer perceptron (MLP) method in [66]; LSTM was the best, and logistic regression ranked second. The forward target window was chosen to be 30 days. Three groups of time-dependent predictor data were used. These groups were designated “fixed time” for demographic data, diagnostic or episodic for biomarker and medical examination data, and “high resolution” for vital signs data monitored on a frequent basis. Their method can be analogous to the previously discussed data fusion method.

#### 5.5.2. Machine Learning for CHFS Prognosis

Two hundred and two statistical models were compared with 78 machine learning models in [78]. Random forests, support vector machine boosting, decision tree, MLP, and deep learning were listed among the machine learning methods. The authors concluded that ML models do not achieve significant benefits in event prediction. On the other hand, the authors of another comparison [119] concluded that machine learning classifiers perform better, but noted that ML prediction models should, as a rule, be reviewed using clinical modeling quality standards.

With a cohort of 30,687 adults, the performance of MLP, random forest, and XGBoost machine learning algorithms was compared with logistic regression in [120]. AUROC values were compared for 30-day, 90-day, and 365-day predictions for four different predictor engineering approaches. Except for the 90-day readmission, the XGBoost predictive models performed better than the other models. Prediction of CHFS 30-day readmission using LSTM was reported in [121].

To conclude this subsection, we would like to add that the aforementioned work [78] also contains a critical comprehensive appreciation of machine learning efforts in CHFS modeling. Using the PROBAST tool, the survey authors concluded that currently, machine learning models generally have poorer clinical feasibility and reliability compared to statistical models.

## 6. Future Directions

The field of heart failure prediction research contains a number of publications that differ in prediction objectives and the nature of input data and their processing. The most elaborated is the prognostic prediction of patient death based on cross-sectional input data, and it is a procedure ready for clinical deployment.

The area of recurrent diagnostics, where the patient is monitored repeatedly and frequently over time, appears to be the least theoretically and practically investigated. The digitization and lowering of the price of medical devices together with the development of telecommunication technologies enables the acquisition of medical information in the patient’s home environment. From this point of view, the methods of recurrent diagnostics deserve attention.

Another suggestion is that the vast majority of prediction publications, at least in the field of CHFS, focus on the prediction of adverse events such as hospital admission or death. In our opinion, the priority should be shifted to the decision-making process of the therapist. In the field of telemedical CHFS, this could lead to optimization of decision-making when administering diuretics or ambulatory up-titration.

As mentioned earlier, there are significant differences between the US and European heart failure guidelines. We believe that the research on disease-specific models developed using UML will be helpful in addressing this issue.

Finally, the machine learning community appears to be accepting challenges from medicine statisticians and has started to accept the rigor and prudence of medicine modeling guidelines. Publications are emerging that directly compare machine learning techniques with well-established statistical methods such as logistic regression. Once these challenges are met, machine learning can become a reliable clinical prediction technology.

## 7. Summary and Conclusions

We believe that we succeeded in achieving the objectives formulated in Section 1. In Section 2, the article provides basic information about CHFS disease, telemedicine and prediction models in general. We also designed or formulated a disease-specific model pair for prognosis and diagnosis in a telemedicine-like setting. The prognostic part of the pair is a discrete health-state model, the diagnostic part has the form of an accepted representation of the long-term progression of heart failure. In Section 4, we addressed the demanding problem of model dimensionality, especially in relation to the various inclusions of time. In addition, we have listed the groups of predictors and other factors determining the prediction performance and explored different approaches to increase the performance of prediction models. The listed characteristics could also serve to classify the models reviewed in Section 5. This section is a structured review of statistical and machine learning prediction models, where researchers have dealt with different prediction situations using a variety of models with an emphasis on chronic heart failure syndrome and recurrent diagnostics. We hope that all the above parts will help researchers who are starting, modifying, or completely re-engineering their clinical prediction models or clinical trial designs.

During our cross-sectional search for articles, we tried not to limit ourselves to a particular research group or direction of predictive research. Current prediction research appears to consist of four distinct research communities, each with slightly different methods and terminology. The first community focuses primarily on the engineering aspects of ADHF detection and diagnostics. The second appears to be made up of medical statisticians who use well-established, especially prognostic prediction methods to maximize benefit to the medical community. The third uses advanced statistical methods to develop a patient prognosis with maximum use of time-dependent medical parameters. The last community, the machine learning community, tends to apply machine learning methods to the detection and diagnosis as well as prognosis of ADHF way similar to that used by the groups mentioned above.

A search based on a single, albeit well-studied, disease has its limitations in addition to its advantages. Recurrently performed remote monitoring, which is part of heart failure syndrome care, revealed to us current efforts to apply new advanced prediction methods. On the other hand, some directions seem to be marginal in the field of heart failure syndrome prediction, and pointing them out is one of the suggestions of our work for the prediction research community.

## 8. Limitations of this Study

Given the primary purpose of providing a brief insight into the current state of clinical predictive modeling, we are aware of several limitations of this work. In the article, we did not explicitly deal with the process of training, validation and calibration of the prediction models. We also did not address the relation of the prediction models to analytical therapeutic processes and decision-making therapeutic processes. For the purposes of the study, clinical information on heart failure syndrome was collected from both European and US guidelines [6,7]. The guidelines differ from each other, so we refer to them frequently in the text to avoid misunderstandings. The study was not written by a medical specialist.

## Figures and Tables

**Table 1:** Different data groups in a telemedical CHFS trial.

Data Group	Temporal Characteristics
Demographic and comorbidity data (baseline)	Not changing during the trial
Entry examination data (baseline)	Collected at the time of entry examination
Signs and symptoms data (telemedicine data)	Time dependent (high repetitive rate)
Episodical or regular visit data	Time dependent (low repetitive rate)
Final examination data	Collected at the time of final examination
Risk-changing clinical milestones *	Episodic or in the patient’s clinical history
Drug dosing and other therapeutic actions	Episodic or in the patient’s clinical history

* The term is used, e.g., in [60].

**Table 2:** Data types structured into groups for the data fusion method.

Entry Examination (Baseline Data) *	Signs and Symptoms (Highly Repetitive Data)
NYHA II–IV	Heart rate
LVEF	Systolic blood pressure
ECG	Diastolic blood pressure
Haemoglobin	Body weight
Serum sodium	Oxygen saturation
Serum potassium	Symptom intensity level
Serum creatine	
NT-proBNP	**Demographics (baseline data)**
CRP	Age
BUN	Race
KCCQ-12	Gender
6-min walk test	

* With the patient home diagnostic box, some biomarkers can be available on a daily basis [65].

## Data Availability

No new data were created or analyzed in this study.

## Abbreviations

The following abbreviations are used in this manuscript:

EHR	Electronic Health Record
TM	Telemedicine
UML	Unified Modeling Language
HF	Heart Failure
CHFS	Chronic Heart Failure Syndrome
ADHF	Acute Decompensation of Heart Failure
SHFM	Seattle Heart Failure Model
ER	Emergency Room
ICU	Intensive Care Unit
MC	Markov Chain
BBN	Bayesian Belief Network
AUROC	Area Under the Receiver Operating Characteristics
ML	Machine Learning
MLP	Multi-Layer Perceptron
LSTM	Long Short-Term Memory
XGBoost	eXtreme Gradient Boosting

## Appendix A. Determination of Coefficients in Cox Regression Using the Maximum Likelihood Estimation Method

Cox’s *partial likelihood function* L(β) is used to determine the values of the β coefficients in the Equation (2). The patient leaves the clinical trial at time $t_{i}$ either because of the occurrence of the investigated event or for another reason usually included under the term censoring. The coefficients β are determined by maximizing the value L(β) whose logarithm is given by [50]

$$\log L(\beta )=\sum_{i=1}^{n}\delta_{i}\left\{\sum_{j=1}^{p}\beta_{j}x_{ji}\left(t_{i}\right)-\log\sum_{l\in R(t_{i})}\exp\left(\sum_{j=1}^{p}\beta_{j}x_{jl}\left(t_{i}\right)\right)\right\}\;,$$

where $\delta_{i}$ takes on values of zero for censored patients and unity for a patient experiencing the investigated event. We could see that if the *i*th patient is censored, $\delta_{i}$ nullifies its contribution in overall summation. Censored patient data values are only partially used in the internal sum and are accounted for through index *l*. The inner sum applies to all patients in subset $R(t_{i})$, which is the subset composed of patients who did not experience an event and were uncensored just before time $t_{i}$ [50]. See [50,51,52] for more details.

## Appendix B. Explanatory Information for the Bayes’ Theorem

The clinical interpretation of the Bayes’ theorem in the form of Equation (3) has four different presentations depending on the state of the variables disease state *D* and test result $E_{1}$. The left side of the equation can have a presentationin that in clinical practice is called *positive predictive value* [82,83]. We can construct an explanatory table for all combinations of *D* and $E_{1}$ in the form of Table A1.

**Table A1:** Explanatory descriptions of Bayes’ theorem terms. D=1 means the presence of the disease, D=0 means the absence of the disease. $E_{1}=1$ means a positive test result and $E_{1}=0$ means a negative test result.

	*D* = 1, $E_{1}$ = 1	$D=0,E_{1}=1$	$D=1,E_{1}=0$	$D=0,E_{1}=0$
$P_{1}\left(D\right|E_{1})$	Positive Predictive Value	False Omission Rate *	False Discovery Rate *	Negative Predictive Value
$P_{0}\left(D\right)$	Prevalence	1 − Prevalence	Prevalence	1 − Prevalence
$P_{0}\left(\neg D\right)$	1 − Prevalence	Prevalence	1 − Prevalence	Prevalence
$P_{1}\left(D\right|E_{1})$	Positive Predictive Value	False Omission Rate *	False Discovery Rate *	Negative Predictive Value
$P(E_{1}|D)$	Sensitivity	1 − Specificity	1 − Sensitivity	Specificity
$P(E_{1}|\neg D)$	1 − Sensitivity	Sensitivity	Specificity	1 − Specificity
$P\left(E_{1}|D\right)P_{0}\left(D\right)$	Probability of true positive	Probability of false positive	Probability of false negative	Probability of true negative
$P\left(E_{1}|\neg D\right)P_{0}\left(\neg D\right)$	Probability of false positive	Probability of true positive	Probability of true negative	Probability of false negative

* It seems, that these terms has not been estabilished in clinical practice yet.

Supplementary information to the Table A1 is given in the Table A2. Definitions of established medical and statistical terms were taken from the basic literature.

**Table A2:** Quantitative definitions of key diagnostic concepts.

Concept Name	Definition
Prevalence	Proportion of a defined group in the population having a disease at one point in time
Sensitivity	Rate of positive responses in a test from persons with a specific disease, true positive rate
Specificity	Rate of negative responses in a test from persons free from a disease, true negative rate
True positives	Number of cases in population correctly identified as diseased
False positives	Number of cases in population incorrectly identified as diseased, type I error
True negatives	Number of cases in population correctly identified as healthy
False negatives	Number of cases in population incorrectly identified as healthy, type II error
